# The Outcome of Neoadjuvant Imatinib Therapy Combined With Surgery for Rectal Gastrointestinal Stromal Tumors: A Report of Three Cases and a Review of the Literature

**DOI:** 10.7759/cureus.12100

**Published:** 2020-12-15

**Authors:** Abdelbassir Ramdani, Tariq Bouhout, Badr Serji, Wafaa Khannoussi, Tijani El Harroudi

**Affiliations:** 1 Surgical Oncology, Regional Oncology Center, Mohammed VI University Hospital, Oujda, MAR; 2 Gastroenterology and Hepatology, Mohammed VI University Hospital Center/Mohammed First University, Oujda, MAR

**Keywords:** rectum, gist, abdominoperineal resection, imatinib, dog1, cd117

## Abstract

Gastrointestinal stromal tumors (GISTs) represent the most frequent mesenchymal tumors of the gastrointestinal tract. They occur most frequently in the stomach. Rectal localization remains rare and represents only 5% of all GIST cases and 0.1% of all rectal tumors. Immunohistochemical staining (CD117, DOG1) and molecular analysis remain the gold standard for diagnosis; DOG1 represents a very sensitive marker regardless of CD117 expression. Complete en-bloc resection constitutes the only curative treatment; however, surgical management of rectal GIST remains challenging and can involve extensive surgery such as abdominoperineal resection with significant morbidity. The role of neoadjuvant Imatinib therapy in rectal GISTs is controversial and mainly indicated in a locally advanced tumor or sphincter invasion to increase the chance of complete resection and sphincter preservation. Herein, we report three cases of a rectal GIST treated with neoadjuvant Imatinib therapy and who underwent extensive surgery with complete resection (R0), as well as a recent review of the literature, to study clinicopathological features, surgical challenges, and perioperative Imatinib therapy outcome of rectal GISTs.

## Introduction

Gastrointestinal stromal tumors (GISTs) are mesenchymal tumors originating from the interstitial cells of Cajal or their precursors. They are located in the stomach and small intestine in the majority of cases. The rectal localization remains exceptional and represents only 5% of all GIST cases and 0.1% of all rectal tumors [1,2]. Complete resection is the gold-standard treatment of GISTs. However, surgical management is challenging and possibly involves extensive and mutilating surgery with anal sphincter sacrifice [3]. The discovery of tyrosine kinase inhibitors such as Imatinib has changed the management of GISTs; neoadjuvant Imatinib therapy seems promising to increase the probability of complete resection and preserving the sphincter [3]. Herein, we describe our experience with three patients treated with neoadjuvant Imatinib therapy and who then underwent extensive surgery with anal sphincter sacrifice; we also refer to a recent review of the literature to discuss clinicopathological features, surgical challenges, and perioperative Imatinib therapy outcome of rectal GISTs.

## Case presentation

Case 1

A 39-year-old married woman and mother of four children presented with complaints of tenesmus and anal pain without any history of rectal bleeding. The digital rectal examination revealed a rigid, immobile, and painful right semi-circumferential rectal mass at 2 cm from the anal verge.

**Figure 1:**
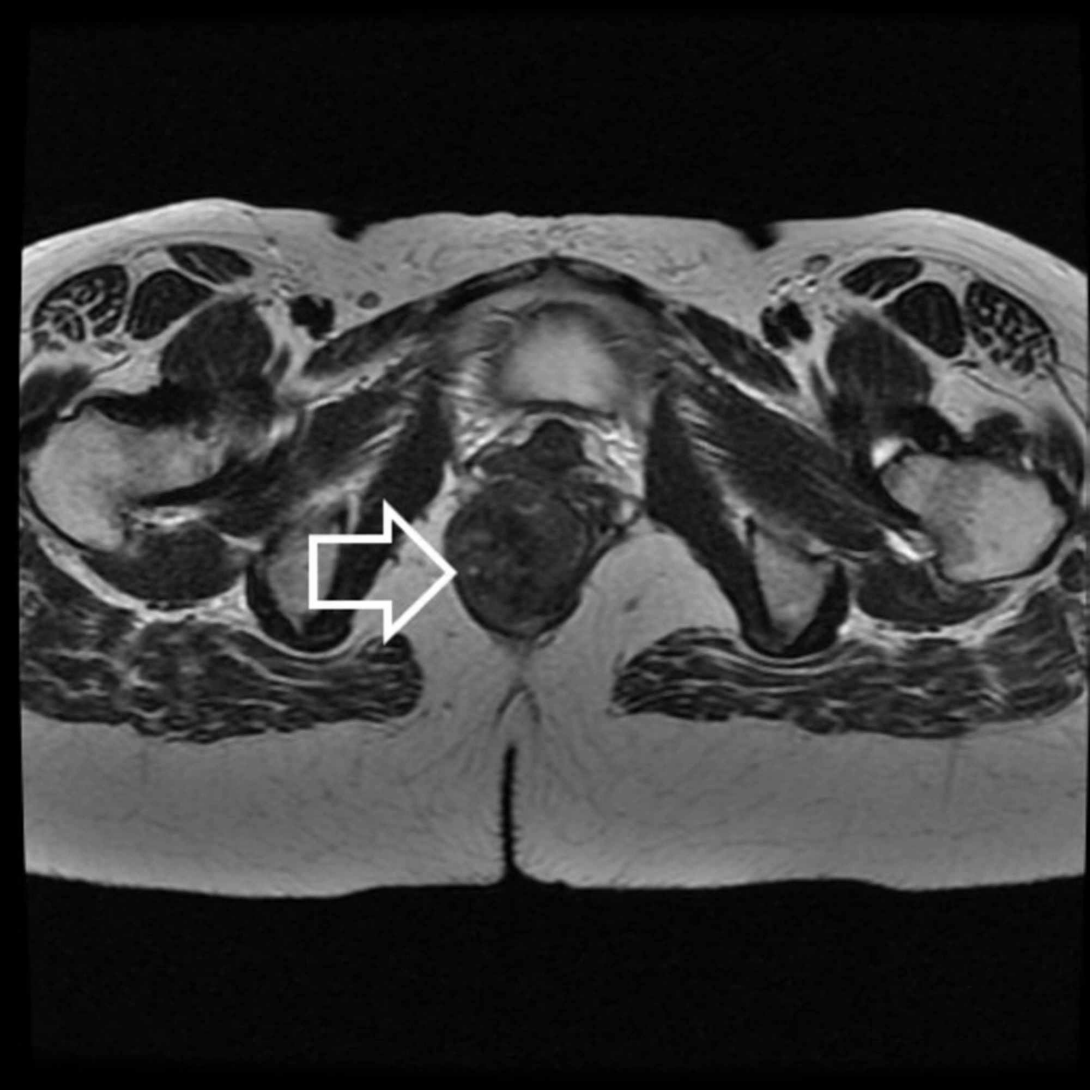
Axial T2-weighted MRI showing a mass of the right later wall of the lower rectum (white arrow)

A colonoscopy concluded the presence of right semi-circumferential rectal mass extending up to 3 cm from the anal verge with regular surface bulging in the rectal lumen. Pelvic magnetic resonance imaging (MRI) revealed a submucosal lesion in the right lateral wall of the lower rectum extending to the anal canal bulging into the rectal and anal lumen with exophytic development with close contact to the external sphincter without invasion or infiltration of adjacent organs (Figure 1).

**Figure 2:**
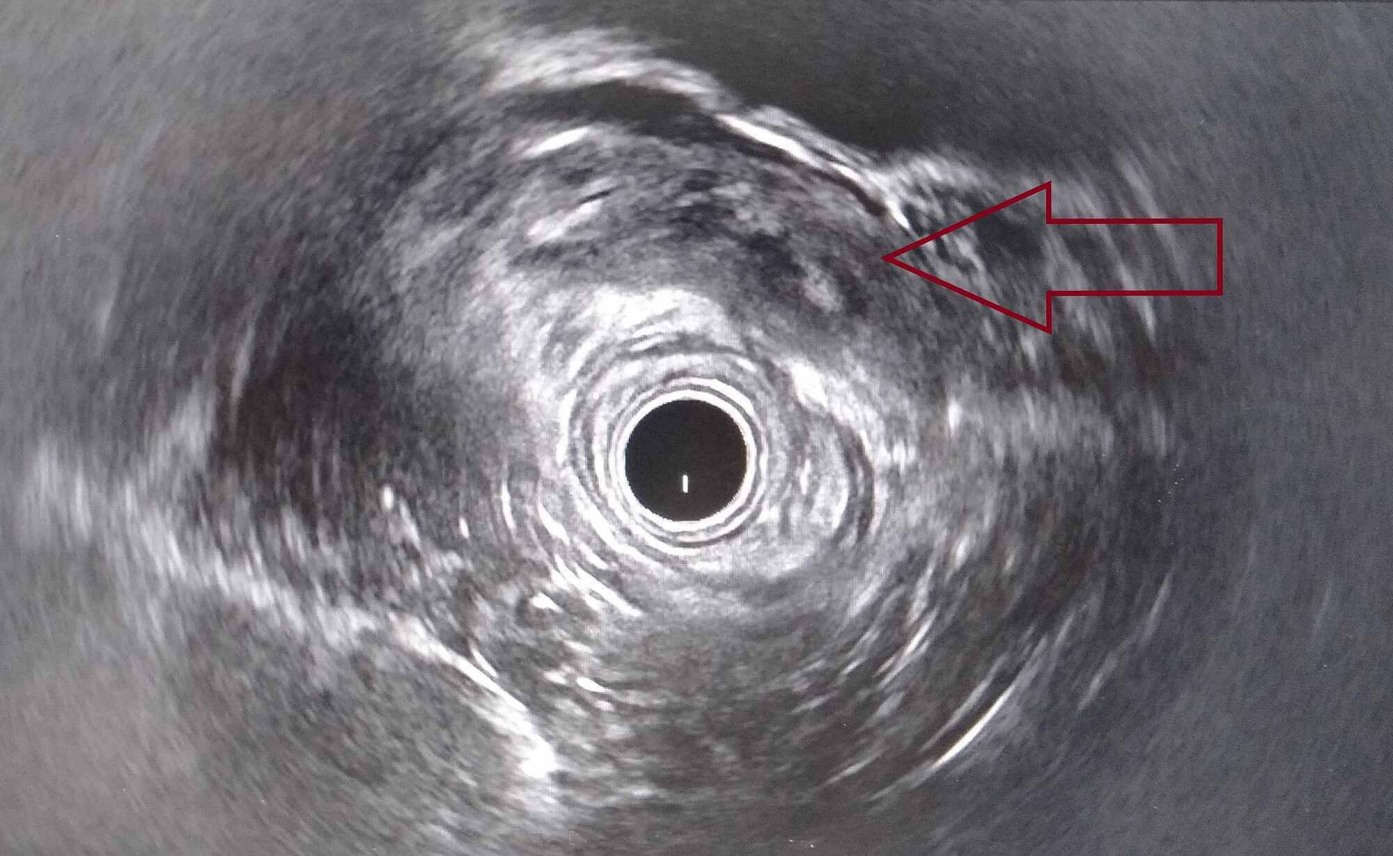
Rectal endoscopic ultrasound showing a well-limited, hypoechoic, and heterogeneous mass of the right lateral rectal wall (red arrow)

Rectal endoscopic ultrasound showed a well-limited, hypoechoic, heterogeneous mass of the right lateral rectal wall, developing at the expense of the submucosa (Figure 2).

**Figure 3:**
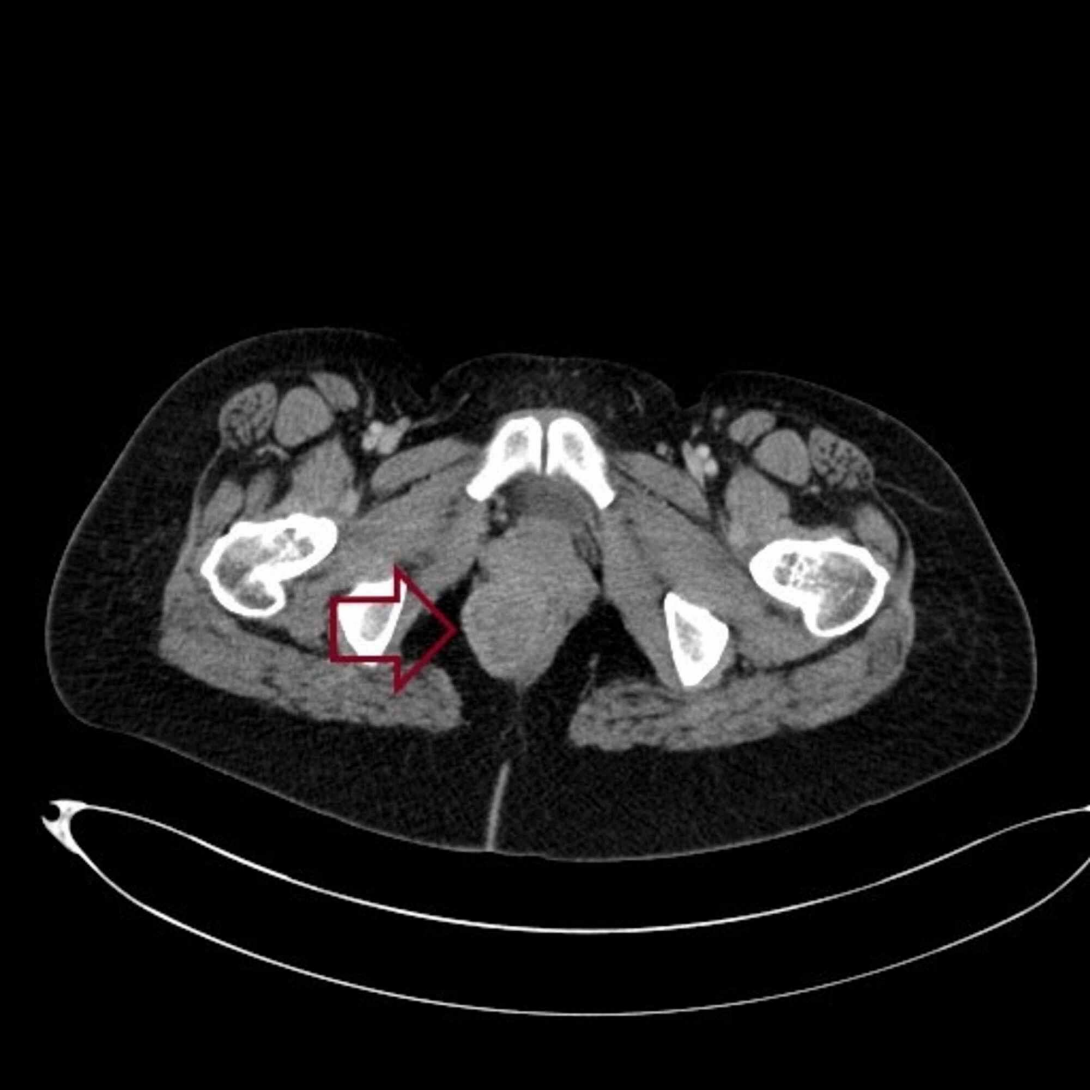
Pelvic computed tomography showing a mass of the right lateral rectal wall (red arrow)

Transrectal needle biopsy with immunohistochemical study confirmed the diagnosis of rectal GIST (CD117-, DOG1+). Thoracic and abdominopelvic computed tomography (CT) showed a mass of the right lateral rectal wall measuring 6 cm, with no distant metastases (Figure 3).

Following a discussion in a multi-disciplinary team (MDT) meeting, she was started on Imatinib at 400 mg once daily for six months. Repeated CT scan showed a stationary aspect of tumor size with no therapeutic response to Imatinib. Due to the expansion of the tumor in the anal canal and resistance to Imatinib, the patient underwent abdominoperineal resection with pseudo-continent perineal colostomy.

**Figure 4:**
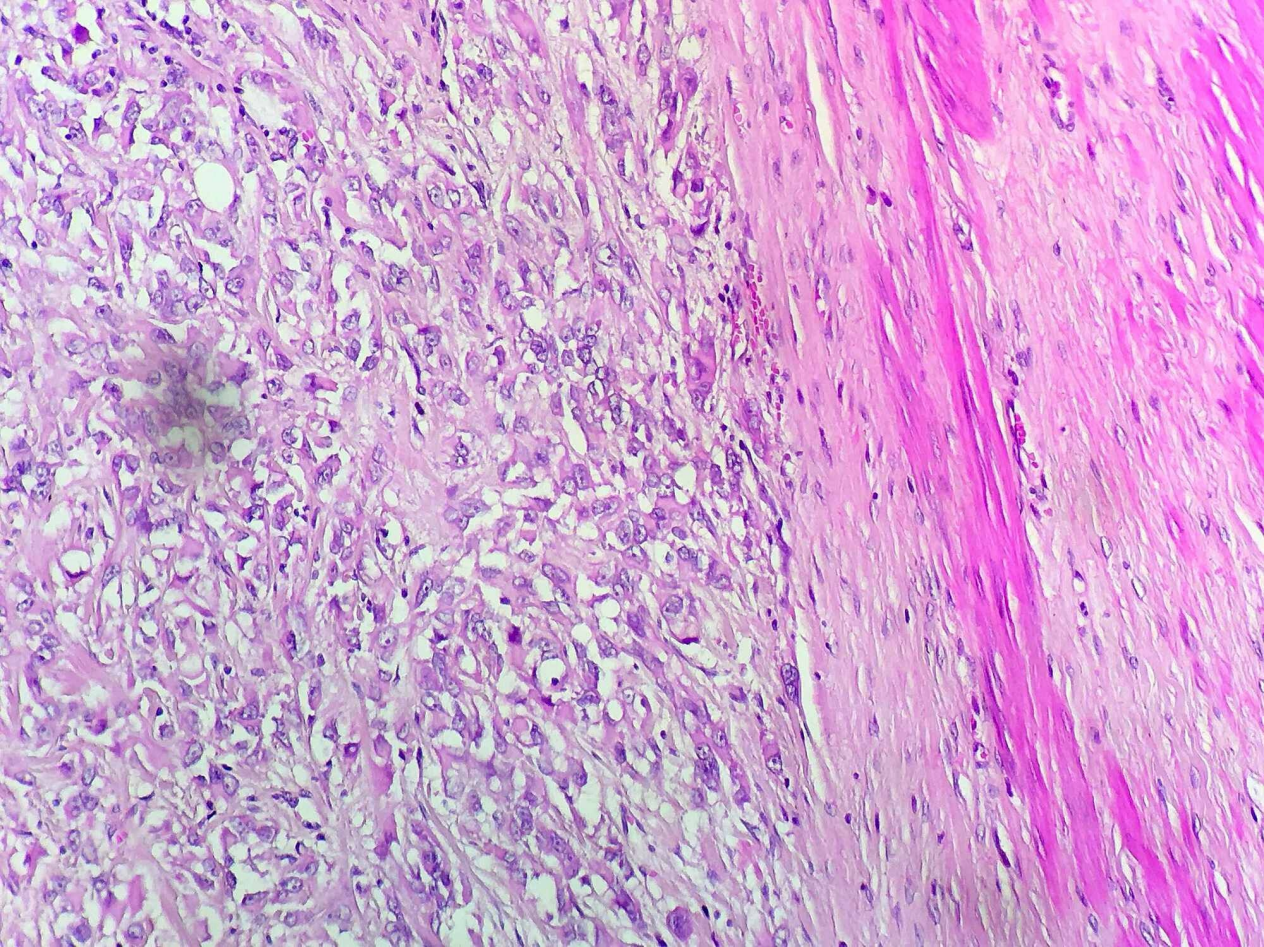
Microphotograph showing epithelioid cells with marked anisokaryosis and irregular nuclei (HE 400x) HE: hematoxylin and eosin

**Figure 5:**
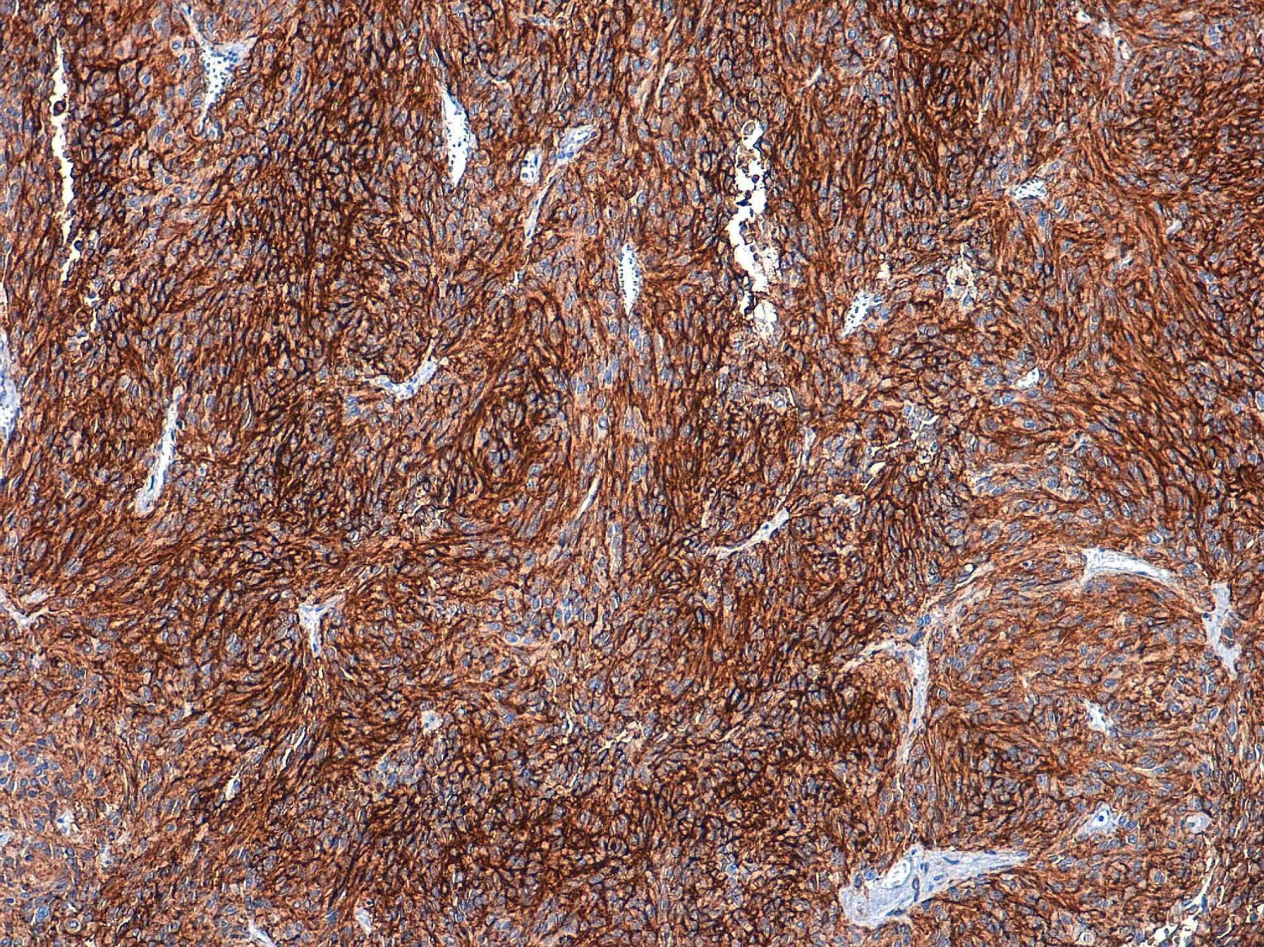
Immunohistochemical stainings showing a cytoplasmic expression of tumor cells by DOG1

The histopathological examination confirmed the diagnosis of rectal GIST measuring 6.5 cm, with a mitotic rate of 2 per 50 high power fields (HPF) classified at high risk of recurrence according to Miettinen’s classification; immunohistochemical stainings revealed a cytoplasmic expression of rare cells by DOG 1 with an absence of CD117 expression (Figures 4, 5).

The postoperative course was uneventful and the patient was discharged from the hospital on day 7. At present, the patient is alive without recurrence after 18 months of follow-up.

Case 2

A 73-year-old hypertensive lady presented with complaints of anal pain and constipation. The digital rectal examination revealed a hard, fix, and non-tender mass with a smooth surface of the anterior rectal wall 2 cm from the anal verge. Colonoscopic examination showed a submucosal tumor on the anterior wall of the rectum just above the pectinate line. Subsequently, a biopsy with immunohistochemical stains was performed, revealing a rectal GIST positive for CD117 and DOG1.

**Figure 6:**
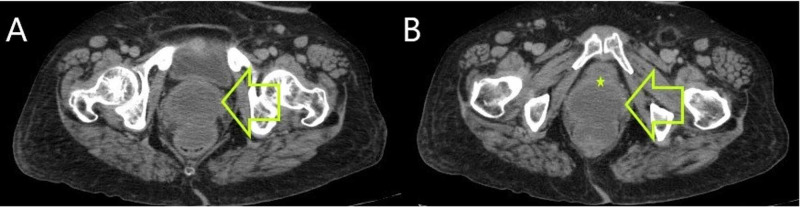
(A and B) Pelvic CT scan showing a mass of the anterior rectal wall (yellow arrows) with an invasion of the posterior vaginal wall (yellow star)

Thoracic and abdominopelvic CT scan revealed a tumor of the anterior lower rectal wall measuring 7 cm with an invasion of the posterior vaginal wall; no distant metastasis was noted (Figures 6A, 6B).

**Figure 7:**
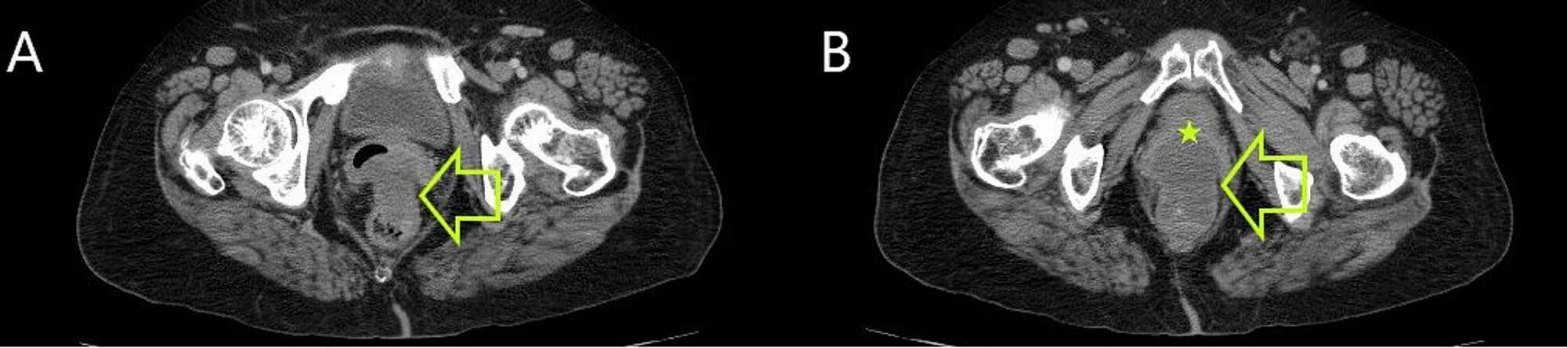
(A and B) Repeated pelvic CT showing a reduction in tumor size of 28.5% (yellow arrows) and an invasion of the posterior vaginal wall (yellow star)

The patient received neoadjuvant Imatinib treatment for one year (400 mg per day) with a marked improvement on the radiological plan, by the reduction in the size of 28.5% (from 7 cm to 5 cm) revealed by a repeated abdominopelvic CT scan (Figures 7A, 7B).

At the end of her treatment, she presented side effects such as bilateral pleural effusion and edema of lower limbs requiring interruption of the treatment. Then she underwent abdominopelvic resection with partial colpectomy and sigmoid colostomy as the distance between the lower edge of the tumor and the upper edge of the sphincter remained unclear.

The histopathological examination confirmed the diagnosis of rectal GIST infiltrating the vaginal wall, measuring 6 cm with a mitotic index of 4 per 50 HPF classified at high risk of recurrence according to Miettinen’s classification. The postoperative course was uneventful, and the patient was discharged from the hospital on day 6. with no recurrence over the 24-month follow-up.

Case 3

A 58-years-old female patient presented with a four-month history of anal pain without other associated signs. The digital rectal examination revealed an immobile and hard mass of the posterior rectal wall. Colonoscopy showed a large submucosal tumor of the posterior wall of the rectum 3 cm from the anal verge. A core needle biopsy with immunohistochemical staining was performed, revealing a rectal GIST positive for CD117 and DOG1.

**Figure 8:**
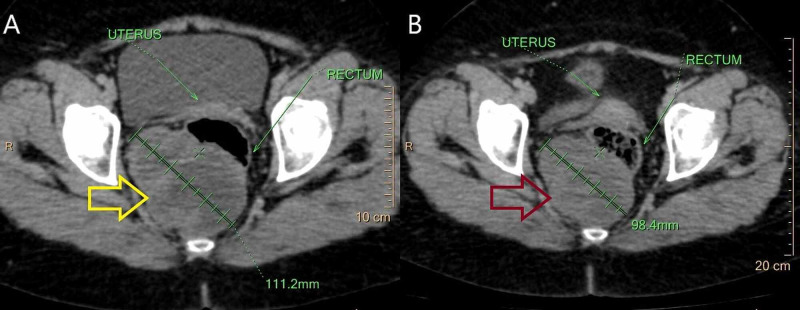
(A) Pelvic CT scan showing a large tumor of the posterior rectal wall (yellow arrow). (B) Repeated pelvic CT scan after one year of neoadjuvant Imatinib therapy showing a reduction in the tumor size of 18.2% (red arrow)

Thoracic and abdominopelvic CT scan showed a tumor of the posterior rectal wall with a heterogeneous enhancement measuring 11 x 9.8 cm, with no distant metastases. Neoadjuvant Imatinib treatment (400 mg/day) was introduced for one year. Repeated CT scan revealed a decrease in tumor size by 18.2% (9 cm vs 11 cm) (Figures 8A, 8B).

As the distance between the upper edge of the sphincter and the inferior pole of the tumor was less than 1 cm, the patient underwent abdominoperineal resection with sigmoid colostomy. Histological examinations and immunohistochemical stains confirmed the diagnosis of rectal GIST measuring 10 cm with a mitotic rate of 5 per 50 HPF classified at high risk of recurrence according to Miettinen’s classification. Imatinib treatment was withheld postoperatively as the patient developed side effects: fatigue and edema of the lower limbs. Currently, she is alive without any evidence of recurrence 36 months after surgery.

Table 1 summarizes the clinicopathological features, perioperative Imatinib therapy outcome, surgical treatment, and the follow-up of our three cases.

**Table 1:** Summary of the three cases HPF: high power fields

	Case 1	Case 2	Case 3
Sex	F	F	F
Age	39	73	58
Symptoms	Tenesmus and anal pain	Constipation and anal pain	Anal pain
Distance from the anal verge	2 cm	2 cm	3 cm
Immunohistochemical stainings	CD117-, DOG1+	CD117+, DOG1+	CD117+, DOG1+
Duration and posology of neoadjuvant Imatinib therapy	Six months (400 mg per day)	One year (400 mg per day)	One year (400 mg per day)
Side effects	None	Bilateral pleural effusion and edema of lower limbs	Fatigue and edema of the lower limbs
Therapeutic response to Imatinib (percentage of tumor size reduction after neoadjuvant Imatinib therapy)	0%	28.50%	18.20%
Type of surgery	Abdominoperineal resection + pseudocontinent perineal colostomy	Abdominoperineal resection + partial colpectomy + sigmoid colostomy	Abdominoperineal resection + sigmoid colostomy
Complete resection (R0)	Yes	Yes	Yes
Mitotic rate	2/50 HPF	4/50 HPF	5/50 HPF
Size	6.5 cm	6 cm	10 cm
Risk of recurrence (Miettinen’s criteria)	High	High	High
Adjuvant Imatinib therapy	No	No	No
Follow-up	18 months	24 months	36 months
Recurrence	No	No	No

## Discussion

GISTs are mesenchymal tumors developing in the majority of cases in the stomach and small intestine; the rectal location remains exceptional with only 5% of cases. The average age at diagnosis is around 60 years according to large European and American cohorts [4,5]. GISTs are generally sporadic except in rare cases associated with familial syndromes such as neurofibromatosis type 1 [6]. The clinical presentation of rectal GIST is varied and nonspecific; the most frequent clinical symptoms include rectal bleeding, rectal mass, change in bowel habits, and abdominal/anal pain [4]. Anal pain was the revealing clinical sign in all our cases.

Colonoscopy remains the first-line complimentary examination; however, the endoscopic findings are not specific; GISTs generally appear like a regular submucosal tumor, with normal or ulcerated mucosa. Endoscopic biopsies are usually negative because the tumor is located in the muscularis propria [3,7]. On rectal endoscopic ultrasound, the tumor presents as a rounded or oval hypoechoic lesion with well-defined margins arising from the hypoechoic fourth layer that corresponds to the muscularis propria [7,8]. Central necrosis, irregular margins, and the presence of intratumoral cystic areas represent signs of malignancy [7].

The indication for a preoperative biopsy should be discussed on a case-by-case basis and remains essential in the event of an unresectable or metastatic tumor or indication of neoadjuvant Imatinib treatment for locally advanced tumors or sphincter invasion requiring an abdominoperineal resection [3,9].

Abdominopelvic and thoracic CT is the gold standard in the GIST extension workup and can detect the invasion of adjacent organs and the presence of liver metastases or peritoneal spread [3,10]. MRI remains more effective in analyzing the locoregional invasion of the tumor; the tumor generally appears hypointense on T1-weighted images, and hyperintense or isointense with high signal intensity areas on T2-weighted images with heterogeneous enhancement after the injection of gadolinium [11].

The fundamental examination to confirm the diagnosis remains the histopathological study with immunohistochemical staining. CD117 is the most important marker with 95% positivity for GISTs. However, it is not specific for GISTs [3]. DOG1 is considered as a sensitive and specific marker of GISTs regardless of CD117 expression; 97.8% of cases expressed DOG1 in a study by West et al. [12]. Liegl et al. reported that 36% of KIT-negative tumors were DOG1 positive, indicating that DOG1 is a more sensitive immunohistochemical marker for GIST than KIT [13]. Other markers are used in the case of CD117 and DOG-1 negativity like CD34, desmine, or protein S100 [3,9]. In our cases, two patients had CD117+; however, patient 1 was KIT negative, and the diagnosis of GIST was made upon DOG1 positivity.

The search for mutations in the KIT and PDGFRA genes made the diagnosis of GIST more accurate; moreover, molecular analysis constitutes, with immunohistochemistry stainings, the gold standard of diagnosis in GIST [14]. The type of mutation influences the prognosis and the effectiveness of adjuvant therapy [3,9]. According to a large European cohort, most of the GISTs were KIT exon 11 mutated (74%); the other mutations found were KIT exon 9 (14%), KIT exon 13 (3%), and only one patient among 156 presented with a PDGFRA mutation; however, 12% of the cases were wildtype with no KIT or PDGFRA gene mutation [4].

Complete en-bloc surgical resection (R0) of the tumor is the only potentially curative treatment [3,9]. The surgical management of rectal GIST is challenging as these tumors are usually large, located in a narrow pelvic space with intimate contact or with possible invasion of pelvic structures, and close to the anal sphincter; complete en-bloc surgical resection may involve extensive surgery with multi-visceral resection or sphincter sacrifice (abdominoperineal resection) with significant morbidity [15]. Intraoperative tumor rupture entails a risk of recurrence by peritoneal dissemination and represents a very pejorative prognostic factor. There is no consensus on the optimal margin for resection. Lymph node dissection is not systematic as the lymphatic spread is rare, and the risk of lymph node recurrence is limited [3]. Lymph node dissection is performed only in the event of macroscopic lymph node involvement. Incomplete resection (R2) represents a poor prognostic factor; however, microscopically incomplete resection (R1) remains subject to discussion because it has not been formally demonstrated that an R1 resection is associated with a worse prognosis [3]. McCarter et al. reported that there was no difference in recurrence-free survival between R0 resection and R1 resection margin [16]; however, according to Zhi et al., R1 resection significantly impacts the disease-free survival and represents a poor prognostic factor [17].

Neoadjuvant Imatinib therapy should be discussed in the MDT meeting for locally advanced tumors or lower rectum GIST requiring abdominoperineal resection to increase the rate of R0 resection and sphincter preservation [3,9]. The evaluation of the effectiveness of treatment must be meticulous to identify patients resistant to Imatinib (5%-10% of cases) for whom surgery is necessary [18]. The duration of 6 to 12 months will achieve an optimal therapeutic rate of 60% to 80% [3]. According to a large European cohort, the median duration of neoadjuvant treatment was 10 months, while maximum tumor reduction was achieved after six months with a median size reduction of 33% [4]. The optimal timing of surgery is still unknown; the attitude proposed by expert surgeons is to perform a CT scan every two or three months and to operate when the tumor is the smallest, or after stability over two consecutive images [18]. Currently, there are no randomized studies of neoadjuvant Imatinib therapy [3].

In our cases, two patients (Case 2 and Case 3) responded partially after one year of neoadjuvant Imatinib therapy with tumor size reduction of 28.5% and 18.2%, respectively; however, no therapeutic response to Imatinib was noted for Case 1 after six months of treatment. Complete resection (R0) was achieved for all cases; however, none of our patients had sphincter preservation after neoadjuvant Imatinib therapy, and all of them underwent extensive surgery. Tielen et al. reported that neoadjuvant Imatinib therapy decreases the tumor size but did not lead to less extensive surgery [19].

The mitotic rate remains the most important prognostic factor for recurrence; Miettinen’s classification (Table 2), which has been used in many recent rectal GIST studies, is a useful tool to estimate the risk of recurrence based on the tumor size and the mitotic rate [15,19,20]. Tumors with a mitotic rate >5 per 50 HPF and/or size >10 cm are at high risk of recurrence; however, tumors <2 cm with a mitotic rate <5 per 50 HPF showed no recurrence in the follow-up [20].

**Table 2:** Miettinen’s classification *Insufficient data due to small number of cases

Mitotic rate	Tumor size	Risk of recurrence
≤5/50 HPF	≤2 cm	None
	>2 - ≤5 cm	Low
	>5 - ≤10 cm	High*
	>10 cm	High
>5/50 HPF	≤2 cm	High
	>2 - ≤5 cm	High
	>5 - ≤10 cm	High*
	>10 cm	High

According to the European Society of Medical Oncology, adjuvant Imatinib therapy is recommended in the case of rectal GIST with a high risk of recurrence for three years or perforated rectal GIST for at least three years [3]. Zhi et al. reported that adjuvant Imatinib treatment could decrease the risk of recurrence for patients with R1 resection [17]. Determination of the tumor genotype is recommended before initiating adjuvant therapy; furthermore, the mutation in PDGFRA exon 18 (D842V) is resistant to Imatinib therapy [3]. All our patients were at high risk of recurrence. Adjuvant Imatinib therapy was not administered to patient 1 due to the Imatinib resistance and the two remaining patients developed side effects requiring the interruption of Imatinib therapy.

According to a French study, overall survival rates at three and five years were 97.5% and 86.5%, respectively. The risk of local and overall relapse was significantly decreased after perioperative Imatinib therapy; moreover, the disease-free survival was improved with no impact on the overall survival [15].

## Conclusions

The rectal localization of GISTs remains exceptional. The diagnosis is made with the histological examination and immunohistochemistry staining. The only curative treatment is complete surgical resection (R0) without intraoperative tumor rupture; however, the surgical management of rectal GISTs remains challenging. In our experience, none of our patients had sphincter preservation after neoadjuvant Imatinib therapy. In the literature, the neoadjuvant Imatinib therapy is indicated mainly in cases of locally advanced tumors or sphincter invasion to increase the probability of complete resection (R0) and sphincter preservation; however, its outcome remains controversial. Randomized studies are necessary to evaluate the efficacy of the neoadjuvant Imatinib therapy.
